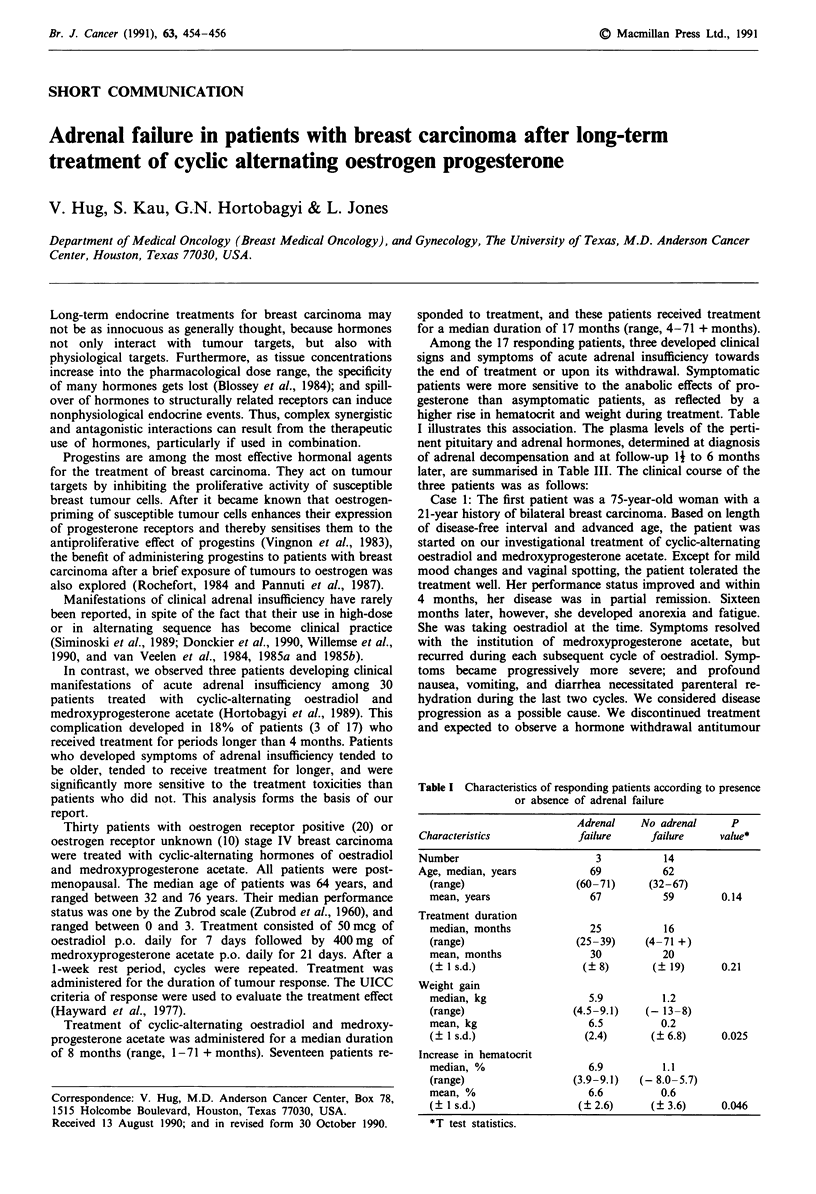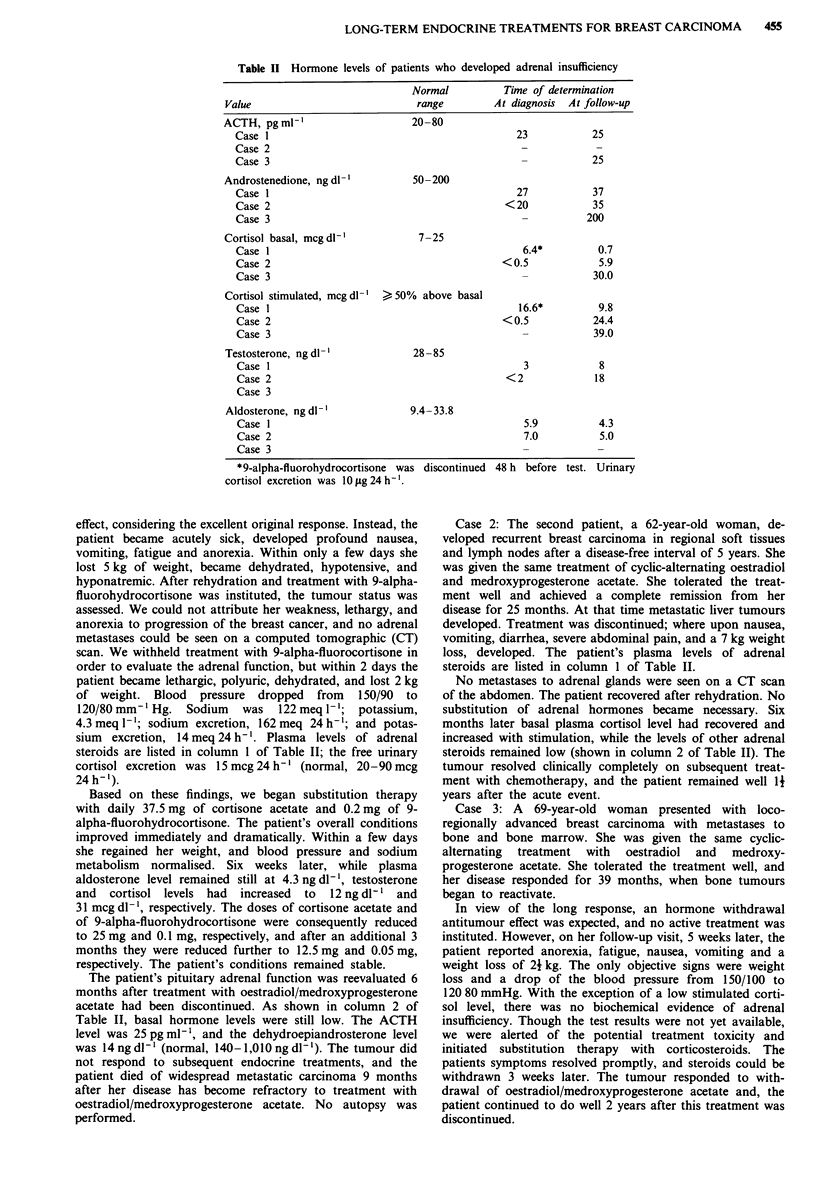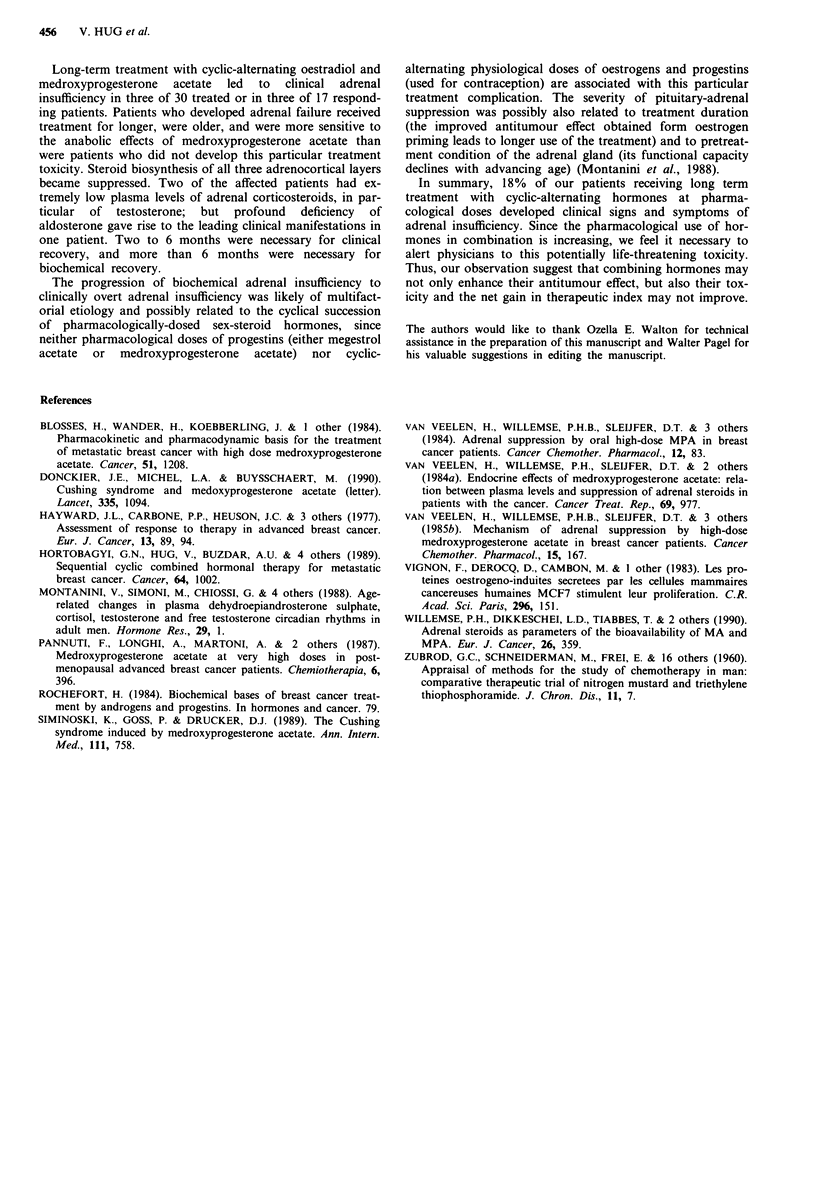# Adrenal failure in patients with breast carcinoma after long-term treatment of cyclic alternating oestrogen progesterone.

**DOI:** 10.1038/bjc.1991.105

**Published:** 1991-03

**Authors:** V. Hug, S. Kau, G. N. Hortobagyi, L. Jones

**Affiliations:** Department of Medical Oncology (Breast Medical Oncology), and Gynecology, University of Texas, M.D. Anderson Cancer Center, Houston 77030.


Br. J. Cancer (1991), 63, 454-456 © Macmillan Press Ltd., 1991

SHORT COMMUNICATION

### Adrenal failure in patients with breast carcinoma after long-term treatment of cyclic alternating oestrogen progesterone

V. Hug, S. Kau, G.N. Hortobagyi & L. Jones

*Department of Medical Oncology (Breast Medical Oncology), and Gynecology, The University of Texas, M.D. Anderson Cancer Center, Houston, Texas 77030, USA.*

Long-term endocrine treatments for breast carcinoma may not be as innocuous as generally thought, because hormones not only interact with tumour targets, but also with physiological targets. Furthermore, as tissue concentrations increase into the pharmacological dose range, the specificity of many hormones gets lost (Blossey *et al.*, 1984); and spill-over of hormones to structurally related receptors can induce nonphysiological endocrine events. Thus, complex synergistic and antagonistic interactions can result from the therapeutic use of hormones, particularly if used in combination.

Progestins are among the most effective hormonal agents for the treatment of breast carcinoma. They act on tumour targets by inhibiting the proliferative activity of susceptible breast tumour cells. After it became known that oestrogen-priming of susceptible tumour cells enhances their expression of progesterone receptors and thereby sensitises them to the antiproliferative effect of progestins (Vingnon *et al.*, 1983), the benefit of administering progestins to patients with breast carcinoma after a brief exposure of tumours to oestrogen was also explored (Rochefort, 1984 and Pannuti *et al.*, 1987).

Manifestations of clinical adrenal insufficiency have rarely been reported, in spite of the fact that their use in high-dose or in alternating sequence has become clinical practice (Siminoski *et al.*, 1989; Donckier *et al.*, 1990, Willemse *et al.*, 1990, and van Veelen *et al.*, 1984, 1985*a* and 1985*b*).

In contrast, we observed three patients developing clinical manifestations of acute adrenal insufficiency among 30 patients treated with cyclic-alternating oestradiol and medroxyprogesterone acetate (Hortobagyi *et al.*, 1989). This complication developed in 18% of patients (3 of 17) who received treatment for periods longer than 4 months. Patients who developed symptoms of adrenal insufficiency tended to be older, tended to receive treatment for longer, and were significantly more sensitive to the treatment toxicities than patients who did not. This analysis forms the basis of our report.

Thirty patients with oestrogen receptor positive (20) or oestrogen receptor unknown (10) stage IV breast carcinoma were treated with cyclic-alternating hormones of oestradiol and medroxyprogesterone acetate. All patients were post-menopausal. The median age of patients was 64 years, and ranged between 32 and 76 years. Their median performance status was one by the Zubrod scale (Zubrod *et al.*, 1960), and ranged between 0 and 3. Treatment consisted of 50 mcg of oestradiol p.o. daily for 7 days followed by 400 mg of medroxyprogesterone acetate p.o. daily for 21 days. After a 1-week rest period, cycles were repeated. Treatment was administered for the duration of tumour response. The UICC criteria of response were used to evaluate the treatment effect (Hayward *et al.*, 1977).

Treatment of cyclic-alternating oestradiol and medroxyprogesterone acetate was administered for a median duration of 8 months (range, 1-71 + months). Seventeen patients responded to treatment, and these patients received treatment for a median duration of 17 months (range, 4-71 + months).

Among the 17 responding patients, three developed clinical signs and symptoms of acute adrenal insufficiency towards the end of treatment or upon its withdrawal. Symptomatic patients were more sensitive to the anabolic effects of progesterone than asymptomatic patients, as reflected by a higher rise in hematocrit and weight during treatment. Table I illustrates this association. The plasma levels of the pertinent pituitary and adrenal hormones, determined at diagnosis of adrenal decompensation and at follow-up 1½ to 6 months later, are summarised in Table III. The clinical course of the three patients was as follows:

Case 1: The first patient was a 75-year-old woman with a 21-year history of bilateral breast carcinoma. Based on length of disease-free interval and advanced age, the patient was started on our investigational treatment of cyclic-alternating oestradiol and medroxyprogesterone acetate. Except for mild mood changes and vaginal spotting, the patient tolerated the treatment well. Her performance status improved and within 4 months, her disease was in partial remission. Sixteen months later, however, she developed anorexia and fatigue. She was taking oestradiol at the time. Symptoms resolved with the institution of medroxyprogesterone acetate, but recurred during each subsequent cycle of oestradiol. Symptoms became progressively more severe; and profound nausea, vomiting, and diarrhea necessitated parenteral re-hydration during the last two cycles. We considered disease progression as a possible cause. We discontinued treatment and expected to observe a hormone withdrawal antitumour

**Table I** Characteristics of responding patients according to presence or absence of adrenal failure

| *Characteristics* | *Adrenal failure* | *No adrenal failure* | *P value** |
|---|---|---|---|
| Number | 3 | 14 | |
| Age, median, years | 69 | 62 | |
| (range) | (60-71) | (32-67) | |
| mean, years | 67 | 59 | 0.14 |
| Treatment duration | | | |
| median, months | 25 | 16 | |
| (range) | (25-39) | (4-71 +) | |
| mean, months | 30 | 20 | |
| (± 1 s.d.) | (± 8) | (± 19) | 0.21 |
| Weight gain | | | |
| median, kg | 5.9 | 1.2 | |
| (range) | (4.5-9.1) | (- 13-8) | |
| mean, kg | 6.5 | 0.2 | |
| (± 1 s.d.) | (2.4) | (± 6.8) | 0.025 |
| Increase in hematocrit | | | |
| median, % | 6.9 | 1.1 | |
| (range) | (3.9-9.1) | (- 8.0-5.7) | |
| mean, % | 6.6 | 0.6 | |
| (± 1 s.d.) | (± 2.6) | (± 3.6) | 0.046 |

*T test statistics.

Correspondence: V. Hug, M.D. Anderson Cancer Center, Box 78, 1515 Holcombe Boulevard, Houston, Texas 77030, USA.
Received 13 August 1990; and in revised form 30 October 1990.

**Table II** Hormone levels of patients who developed adrenal insufficiency

| *Value* | *Normal range* | *Time of determination* *At diagnosis* | *At follow-up* |
|---|---|---|---|
| ACTH, pg $ml^{-1}$ | 20–80 | | |
| Case 1 | | 23 | 25 |
| Case 2 | | – | – |
| Case 3 | | – | 25 |
| Androstenedione, ng $dl^{-1}$ | 50–200 | | |
| Case 1 | | 27 | 37 |
| Case 2 | | <20 | 35 |
| Case 3 | | – | 200 |
| Cortisol basal, mcg $dl^{-1}$ | 7–25 | | |
| Case 1 | | 6.4* | 0.7 |
| Case 2 | | <0.5 | 5.9 |
| Case 3 | | – | 30.0 |
| Cortisol stimulated, mcg $dl^{-1}$ | ⩾50% above basal | | |
| Case 1 | | 16.6* | 9.8 |
| Case 2 | | <0.5 | 24.4 |
| Case 3 | | – | 39.0 |
| Testosterone, ng $dl^{-1}$ | 28–85 | | |
| Case 1 | | 3 | 8 |
| Case 2 | | <2 | 18 |
| Case 3 | | | |
| Aldosterone, ng $dl^{-1}$ | 9.4–33.8 | | |
| Case 1 | | 5.9 | 4.3 |
| Case 2 | | 7.0 | 5.0 |
| Case 3 | | – | – |

*9-alpha-fluorohydrocortisone was discontinued 48 h before test. Urinary cortisol excretion was 10 μg 24 $h^{-1}$.

effect, considering the excellent original response. Instead, the patient became acutely sick, developed profound nausea, vomiting, fatigue and anorexia. Within only a few days she lost 5 kg of weight, became dehydrated, hypotensive, and hyponatremic. After rehydration and treatment with 9-alpha-fluorohydrocortisone was instituted, the tumour status was assessed. We could not attribute her weakness, lethargy, and anorexia to progression of the breast cancer, and no adrenal metastases could be seen on a computed tomographic (CT) scan. We withheld treatment with 9-alpha-fluorocortisone in order to evaluate the adrenal function, but within 2 days the patient became lethargic, polyuric, dehydrated, and lost 2 kg of weight. Blood pressure dropped from 150/90 to 120/80 $mm^{-1}$ Hg. Sodium was 122 meq $l^{-1}$; potassium, 4.3 meq $l^{-1}$; sodium excretion, 162 meq 24 $h^{-1}$; and potassium excretion, 14 meq 24 $h^{-1}$. Plasma levels of adrenal steroids are listed in column 1 of Table II; the free urinary cortisol excretion was 15 mcg 24 $h^{-1}$ (normal, 20–90 mcg 24 $h^{-1}$).

Based on these findings, we began substitution therapy with daily 37.5 mg of cortisone acetate and 0.2 mg of 9-alpha-fluorohydrocortisone. The patient's overall conditions improved immediately and dramatically. Within a few days she regained her weight, and blood pressure and sodium metabolism normalised. Six weeks later, while plasma aldosterone level remained still at 4.3 ng $dl^{-1}$, testosterone and cortisol levels had increased to 12 ng $dl^{-1}$ and 31 mcg $dl^{-1}$, respectively. The doses of cortisone acetate and of 9-alpha-fluorohydrocortisone were consequently reduced to 25 mg and 0.1 mg, respectively, and after an additional 3 months they were reduced further to 12.5 mg and 0.05 mg, respectively. The patient's conditions remained stable.

The patient's pituitary adrenal function was reevaluated 6 months after treatment with oestradiol/medroxyprogesterone acetate had been discontinued. As shown in column 2 of Table II, basal hormone levels were still low. The ACTH level was 25 pg $ml^{-1}$, and the dehydroepiandrosterone level was 14 ng $dl^{-1}$ (normal, 140–1,010 ng $dl^{-1}$). The tumour did not respond to subsequent endocrine treatments, and the patient died of widespread metastatic carcinoma 9 months after her disease has become refractory to treatment with oestradiol/medroxyprogesterone acetate. No autopsy was performed.

Case 2: The second patient, a 62-year-old woman, developed recurrent breast carcinoma in regional soft tissues and lymph nodes after a disease-free interval of 5 years. She was given the same treatment of cyclic-alternating oestradiol and medroxyprogesterone acetate. She tolerated the treatment well and achieved a complete remission from her disease for 25 months. At that time metastatic liver tumours developed. Treatment was discontinued; where upon nausea, vomiting, diarrhea, severe abdominal pain, and a 7 kg weight loss, developed. The patient's plasma levels of adrenal steroids are listed in column 1 of Table II.

No metastases to adrenal glands were seen on a CT scan of the abdomen. The patient recovered after rehydration. No substitution of adrenal hormones became necessary. Six months later basal plasma cortisol level had recovered and increased with stimulation, while the levels of other adrenal steroids remained low (shown in column 2 of Table II). The tumour resolved clinically completely on subsequent treatment with chemotherapy, and the patient remained well 1½ years after the acute event.

Case 3: A 69-year-old woman presented with loco-regionally advanced breast carcinoma with metastases to bone and bone marrow. She was given the same cyclic-alternating treatment with oestradiol and medroxy-progesterone acetate. She tolerated the treatment well, and her disease responded for 39 months, when bone tumours began to reactivate.

In view of the long response, an hormone withdrawal antitumour effect was expected, and no active treatment was instituted. However, on her follow-up visit, 5 weeks later, the patient reported anorexia, fatigue, nausea, vomiting and a weight loss of 2½ kg. The only objective signs were weight loss and a drop of the blood pressure from 150/100 to 120 80 mmHg. With the exception of a low stimulated cortisol level, there was no biochemical evidence of adrenal insufficiency. Though the test results were not yet available, we were alerted of the potential treatment toxicity and initiated substitution therapy with corticosteroids. The patients symptoms resolved promptly, and steroids could be withdrawn 3 weeks later. The tumour responded to withdrawal of oestradiol/medroxyprogesterone acetate and, the patient continued to do well 2 years after this treatment was discontinued.

Long-term treatment with cyclic-alternating oestradiol and medroxyprogesterone acetate led to clinical adrenal insufficiency in three of 30 treated or in three of 17 responding patients. Patients who developed adrenal failure received treatment for longer, were older, and were more sensitive to the anabolic effects of medroxyprogesterone acetate than were patients who did not develop this particular treatment toxicity. Steroid biosynthesis of all three adrenocortical layers became suppressed. Two of the affected patients had extremely low plasma levels of adrenal corticosteroids, in particular of testosterone; but profound deficiency of aldosterone gave rise to the leading clinical manifestations in one patient. Two to 6 months were necessary for clinical recovery, and more than 6 months were necessary for biochemical recovery.

The progression of biochemical adrenal insufficiency to clinically overt adrenal insufficiency was likely of multifactorial etiology and possibly related to the cyclical succession of pharmacologically-dosed sex-steroid hormones, since neither pharmacological doses of progestins (either megestrol acetate or medroxyprogesterone acetate) nor cyclic-alternating physiological doses of oestrogens and progestins (used for contraception) are associated with this particular treatment complication. The severity of pituitary-adrenal suppression was possibly also related to treatment duration (the improved antitumour effect obtained form oestrogen priming leads to longer use of the treatment) and to pretreatment condition of the adrenal gland (its functional capacity declines with advancing age) (Montanini *et al.*, 1988).

In summary, 18% of our patients receiving long term treatment with cyclic-alternating hormones at pharmacological doses developed clinical signs and symptoms of adrenal insufficiency. Since the pharmacological use of hormones in combination is increasing, we feel it necessary to alert physicians to this potentially life-threatening toxicity. Thus, our observation suggest that combining hormones may not only enhance their antitumour effect, but also their toxicity and the net gain in therapeutic index may not improve.

The authors would like to thank Ozella E. Walton for technical assistance in the preparation of this manuscript and Walter Pagel for his valuable suggestions in editing the manuscript.

### References

BLOSSES, H., WANDER, H., KOEBBERLING, J. & 1 other (1984). Pharmacokinetic and pharmacodynamic basis for the treatment of metastatic breast cancer with high dose medroxyprogesterone acetate. *Cancer*, **51**, 1208.

DONCKIER, J.E., MICHEL, L.A. & BUYSSCHAERT, M. (1990). Cushing syndrome and medoxyprogesterone acetate (letter). *Lancet*, **335**, 1094.

HAYWARD, J.L., CARBONE, P.P., HEUSON, J.C. & 3 others (1977). Assessment of response to therapy in advanced breast cancer. *Eur. J. Cancer*, **13**, 89, 94.

HORTOBAGYI, G.N., HUG, V., BUZDAR, A.U. & 4 others (1989). Sequential cyclic combined hormonal therapy for metastatic breast cancer. *Cancer*, **64**, 1002.

MONTANINI, V., SIMONI, M., CHIOSSI, G. & 4 others (1988). Age-related changes in plasma dehydroepiandrosterone sulphate, cortisol, testosterone and free testosterone circadian rhythms in adult men. *Hormone Res.*, **29**, 1.

PANNUTI, F., LONGHI, A., MARTONI, A. & 2 others (1987). Medroxyprogesterone acetate at very high doses in postmenopausal advanced breast cancer patients. *Chemiotherapia*, **6**, 396.

ROCHEFORT, H. (1984). Biochemical bases of breast cancer treatment by androgens and progestins. In hormones and cancer. 79.

SIMINOSKI, K., GOSS, P. & DRUCKER, D.J. (1989). The Cushing syndrome induced by medroxyprogesterone acetate. *Ann. Intern. Med.*, **111**, 758.

VAN VEELEN, H., WILLEMSE, P.H.B., SLEIJFER, D.T. & 3 others (1984). Adrenal suppression by oral high-dose MPA in breast cancer patients. *Cancer Chemother. Pharmacol.*, **12**, 83.

VAN VEELEN, H., WILLEMSE, P.H., SLEIJFER, D.T. & 2 others (1984*a*). Endocrine effects of medroxyprogesterone acetate: relation between plasma levels and suppression of adrenal steroids in patients with the cancer. *Cancer Treat. Rep.*, **69**, 977.

VAN VEELEN, H., WILLEMSE, P.H.B., SLEIJFER, D.T. & 3 others (1985*b*). Mechanism of adrenal suppression by high-dose medroxyprogesterone acetate in breast cancer patients. *Cancer Chemother. Pharmacol.*, **15**, 167.

VIGNON, F., DEROCQ, D., CAMBON, M. & 1 other (1983). Les proteines oestrogeno-induites secretees par les cellules mammaires cancereuses humaines MCF7 stimulent leur proliferation. *C.R. Acad. Sci. Paris*, **296**, 151.

WILLEMSE, P.H., DIKKESCHEI, L.D., TIABBES, T. & 2 others (1990). Adrenal steroids as parameters of the bioavailability of MA and MPA. *Eur. J. Cancer*, **26**, 359.

ZUBROD, G.C., SCHNEIDERMAN, M., FREI, E. & 16 others (1960). Appraisal of methods for the study of chemotherapy in man: comparative therapeutic trial of nitrogen mustard and triethylene thiophosphoramide. *J. Chron. Dis.*, **11**, 7.